# Optimization of the Biosynthesis of B-Ring *Ortho*-Hydroxy Lated Flavonoids Using the 4-Hydroxyphenylacetate 3-Hydroxylase Complex (HpaBC) of *Escherichia coli*

**DOI:** 10.3390/molecules26102919

**Published:** 2021-05-14

**Authors:** Longji Wang, Xiubing Ma, Haixiang Ruan, Yang Chen, Liping Gao, Ting Lei, Yan Li, Lin Gui, Lina Guo, Tao Xia, Yunsheng Wang

**Affiliations:** 1 School of Life Science, Anhui Agricultural University, Hefei 230036, China; wlj18326662118@163.com (L.W.); 18855112725@163.com (X.M.); 13645516418@163.com (H.R.); chenyangshouji2hao@126.com (Y.C.); gaolp62@126.com (L.G.); 18755195565@163.com (T.L.); liyantea@126.com (Y.L.); lin.gui@anlongen.com (L.G.); gln2570874851@163.com (L.G.); 2 State Key Laboratory of Tea Plant Biology and Utilization, Anhui Agricultural University, Hefei 230036, China; xiatao62@126.com

**Keywords:** B-ring *ortho*-hydroxylation, *Escherichia coli*, flavonoids, 4-hydroxyphenylacetate 3-hydroxylase, biosynthesis

## Abstract

Flavonoids are important plant metabolites that exhibit a wide range of physiological and pharmaceutical functions. Because of their wide biological activities, such as anti-inflammatory, antioxidant, antiaging and anticancer, they have been widely used in foods, nutraceutical and pharmaceuticals industries. Here, the hydroxylase complex *HpaBC* was selected for the efficient in vivo production of *ortho*-hydroxylated flavonoids. Several *HpaBC* expression vectors were constructed, and the corresponding products were successfully detected by feeding naringenin to vector-carrying strains. However, when *HpaC* was linked with an S-Tag on the C terminus, the enzyme activity was significantly affected. The optimal culture conditions were determined, including a substrate concentration of 80 mg·L^−1^, an induction temperature of 28 °C, an M9 medium, and a substrate delay time of 6 h after IPTG induction. Finally, the efficiency of eriodictyol conversion from P2&3-carrying strains fed naringin was up to 57.67 ± 3.36%. The same strategy was used to produce catechin and caffeic acid, and the highest conversion efficiencies were 35.2 ± 3.14 and 32.93 ± 2.01%, respectively. In this paper, the catalytic activity of *HpaBC* on dihydrokaempferol and kaempferol was demonstrated for the first time. This study demonstrates a feasible method for efficiently synthesizing in vivo B-ring dihydroxylated flavonoids, such as catechins, flavanols, dihydroflavonols and flavonols, in a bacterial expression system.

## 1. Introduction

Flavonoids are naturally occurring important secondary metabolites predominantly originating from plants and fungi, and they display diverse bioactivities and distinguished application potential [1,2]. Several studies have shown that these metabolites possess significant pharmacological activities, such as antioxidant [3], antimutagenic [4], anticarcinogenic [5] and antibacterial [6] properties.

Chemically, the skeletal structure of flavonoids has 15 carbons, which consists of two phenyl rings (A and B) and a heterocyclic ring (C). Naturally, flavonoids are plant-derived products and they are found in different parts of the plants, which comprise of six major subgroups, including chalcones, flavones, flavonols, flavan-3-ols, anthocyanins and proanthocyanins [7]. A number of phenolic hydroxy groups of flavonoids are shown to have antioxidant activity, the capacity of free radical scavenging and many other special biological activities. Several studies have indicated that hydroxyl radical scavenging activity is positively correlated with the number of hydroxy groups on ring B; for instance, quercetin (Q, *ortho*-catechol 3′,4′-OH on ring B) has a stronger antioxidant capacity than kaempferol (K, 4′-OH on ring B) [8,9]. Two cytochrome P450-dependent monooxygenases (P450s) in plants, flavonoid 3′-hydroxylase (*F3*′*H*) and flavonoid 3′, 5′-hydroxylase, determined (*F3*′*5*′*H*) the presence and the number of hydroxy groups on the B-ring of flavonoids (*F3*′*5*′*H*) [10,11]. Previous research has demonstrated that *F3*′*H* and *F3*′*5*′*H* can be successfully expressed in a yeast system [12,13,14], whereas these genes are difficult to express in a bacterial expression system. One successful example of the co-expression of a plant *F3*′*5*′*H* and other flavonoid genes in *Escherichia coli* showed that flavonols could be synthesized from phenylpropanoid acids, while the catalytic activity was relatively low [15]. To address this hurdle, much effort has been focused on finding suitable enzyme replacements for the P450-catalyzed hydroxylation of flavonoids.

The 4-hydroxyphenylacetate 3-monooxygenase (*HpaB*) and NAD (P)H-flavin oxidoreductase (*HpaC*) genes from *E. coli* encode the 4-hydroxyphenylacetate 3-hydroxylase complex [16]. It has been suggested that HpaC uses NADH to generate reduced flavin mononucleotides (FMNH^−^), and *HpaB* utilizes FMNH^−^ to catalyze the hydroxylation of phenolic compounds. Previous research has shown that this complex demonstrates gram-scale conversion of a variety of substrates, including *p*-coumaric acid, tyrosol, coniferaldehyde and umbelliferone, to their corresponding *ortho*-hydroxylated counterparts [17]. Further research confirmed the ability of the *HpaBC* hydroxylase complex to convert naringenin (N) and afzelechin (Af) to the corresponding *ortho*-hydroxylated flavonoids [18]. However, the comparison of their catalytic efficiency for different *para*-hydroxylated flavonoid substrates needs further systematic analysis.

In this paper, we have constructed a variety of *HpaBC* expression vectors, and the corresponding products were successfully detected by feeding of N. To increase the conversion efficiency of fermentation products further, we optimized fermentation conditions including medium, induction temperature, substrate concentration and substrate delay time. Ultimately, using optimum conditions, we demonstrated the ability of the *HpaBC* hydroxylase complex to act on *p*-coumaric acid (*p*-CA), N, dihydrokaempferol (DHK), kaempferol (K) and Af to form the corresponding *ortho*-hydroxylated products. This study demonstrated that, using a bacterial expression system, it is feasible to efficiently synthesize *ortho*-hydroxylated flavonoids in vivo, such as catechins, flavanols and flavonols.

## 2. Materials and Methods

### 2.1. Chemicals

Cyanidin (CYA), pelargonidin (PEL), N, eriodictyol (E), K, quercetin (Q), DHK, dihydroquercetin (DHQ), catechin (C), *p*-CA and caffeic acid (CA) were purchased from Shanghai TOT Chemicals Firm (Shanghai, China). Af was purchased from Yuan ye Biotechnology Co., Ltd. All other chemicals were analytical grade chemicals.

### 2.2. Media, Bacterial Strains and Vectors

The media, bacterial strains and vectors used in this study are given in Table 1. The P1 and P2 is pRSFDuet vector and the two genes were inserted with different sites. In the P1 pRSFDuet vector *HpaB* gene is inserted into the first multiple cloning site of the pRSFDuet vector, and the *HpaC* gene is inserted into the second multiple cloning site. Similarly, in the P2 pRSFDuet vector the H*pa*C gene was inserted into the first multiple cloning site, and the H*pa*B gene is inserted into the second multiple cloning site. P3 and P4 is pETDuet vector with different cloning sites. In P3 PETDuet vector, *HpaB* gene is inserted into the first multiple cloning site and the other gene *HpaC* gene is inserted into the second multiple cloning site; in the P4 PETDuet vector the *HpaC* gene is inserted into the first multiple cloning site of the PETdut vector, and the *HpaP* gene is inserted into the second multiple cloning site. The P1 and p2 were transformed into *E. coli* BL21 for co-expression.

LB medium was used for inoculum preparation and protein expression. Modified M9 (M9) medium and Terrific Broth (TB) were used for feeding experiments and de novo production of target compounds. LB medium contained NaCl (1%, *w/v*), tryptone (1.0%, *w/v*) and yeast extract (0.5%, *w/v*) per liter. M9 medium contained glucose (0.4%, *w/v*), Na_2_HPO_4_ (40 mM), NaCl (0.25%, *w/v*), KH_2_PO_4_ (17 mM), NH_4_Cl (19 mM), MgSO_4_ (2 mM), MCaCl_2_ (1 mM) then set volume to 1 L. The one-liter TB liquid medium contained tryptone (1.2%, *w/v*), yeast extract (2.4%, *w/v*), glycerol (0.4%, *v/v*), KH_2_PO_4_ (17 mM), and K_2_HPO_4_ (72 mM). The bacterial strains and plasmids that were used or constructed in this study are listed in Table 1. *E. coli* DH5α was used to propagate all plasmids, while strain BL21 * (DE3) was used as the host for flavonoid production. The vectors pRSFDuet and pETDuet (Novagen) were used as the basis for all plasmid construction and pathway expression.

### 2.3. Construction of the HpaB and HpaC Expression Plasmids

The amplified DNA fragments of *HpaB* and *HpaC* were digested with *Nde* I and *Xho* I and then inserted into multiple cloning site 2 (MCS-2) of the pETDuet or pRSFDuet plasmid. On the basis of these plasmids, we transferred the genes into multiple cloning site 1 (MCS-1) of the pETDuet or pRSFDuet plasmid using a one-step cloning method. The constructed recombinant expression plasmids are shown in Table 1, and the primers used are shown in Table S1. The resulting plasmids were amplified in DH5α cells. The recombinant plasmids were confirmed by sequencing, and these plasmids were introduced into BL21 * (DE3) cells by transformation.

Single colonies were inoculated into 4 mL of LB media containing ampicillin or kanamycin and were cultured overnight at 37 °C. The overnight cultures were inoculated into 10 mL of M9 or TB medium. The cultures were allowed to grow at 37 °C until the optical density at 600 nm (OD_600_) reached 0.6 and then were induced with 1 mM IPTG at 20 °C, 28 °C or 37 °C for 4 h, 6 h or 8 h. The bacterial strains were fed with substrates for *ortho*-hydroxylated flavonoid production. The samples collection was performed at regular time intervals. The OD_600_ was measured for cell growth, and the concentrations of the products and intermediates were analyzed by high-performance liquid chromatography (HPLC) and LC-MS. The products were extracted with ethyl acetate, and all experiments were performed in duplicate.

### 2.4. HPLC and LC-MS Analysis

The HPLC analysis was performed using a C18 column (150 × 4.6 mm i.d.: Luna^®^ 5 µm C18), Phenomenex, Torrance, CA, USA) with an LC-10Avp system (Shimadzu, Kyoto, Japan). The mobile phase comprises of acetonitrile (solvent A) and water (solvent B) (both contained 1% formic acid) at a flow rate of 0.4 mL·min^−1^. The HPLC program was as follows: 10% to 15% B (*v/v*) for 5 min, 15% to 40% B from 5 to 15 min, 40% to 60% B from 20 to 22 min, and 10% B for 22 to 25 min. N, E, K, Q, DHK, DHQ, C and Af were monitored at 280 nm; *p*-CA and CA were monitored at 340 nm; and the anthocyanins were monitored at 530 nm. For further identification of the products, a liquid chromatography mass spectrum (LC-MS) system was used as previously described [19]. The quantitative products of Caffeic acid, Eriodictyol, Catechin, Quercetin and Dihydroquercetin were respectively used and their standard curves were plotted at 280 nm.

### 2.5. Statistical Analysis

Statistical differences were analyzed with SPSS 19.0 using one-way analysis of variance. The results were expressed as the means ± the standard errors of the mean. The error bars represent the standard deviation for at least three replicates.

## 3. Results

### 3.1. Expression of HpaB and HpaC in E. coli

**Figure 1:**
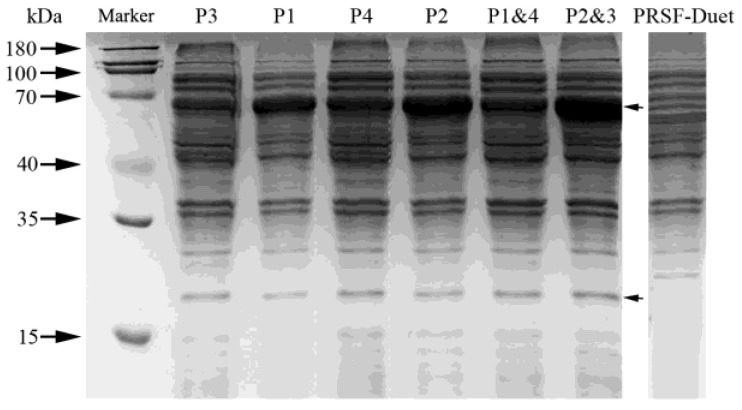
SDS-PAGE of the proteins HpaB and HpaC. The protein expression of different plasmids in BL21 cells. P1: pRSFDuet-HpaBC; P2: pRSFDuet-HpaCB; P3: pETDuet-HpaBC; P4: pETDuet-HpaCB; P2&3: co-expression of P2 and P3; and P1&4: co-expression of P1 and P4. The locations of the HpaB and HpaC proteins are indicated by the arrows on the right. The molecular weights of the marker proteins (180 kDa, 100 kDa, 70 kDa, 40 kDa, 35 kDa and 15 kDa) are also shown.

The open reading frames (ORFs) of *HpaB,* which encodes for the monooxygenase component (GenBank CAD6019151.1), and *HpaC* which encodes for oxidoreductase component (GenBank CAD6019161.1), were cloned into the expression vectors pETDuet and pRSFDuet (Novagen, Carlsbad, CA, USA), respectively. The following results indicate that *HpaB* and *HpaC* were successfully and recombinantly expressed in *E. coli* cells. SDS-PAGE analysis revealed that there were the presence of major bands corresponding to *HpaB* (58.5 kDa) and *HpaC* (18.6 kDa) in samples prepared from the soluble fractions of *E. coli* cells (Figure 1).

**Figure 2:**
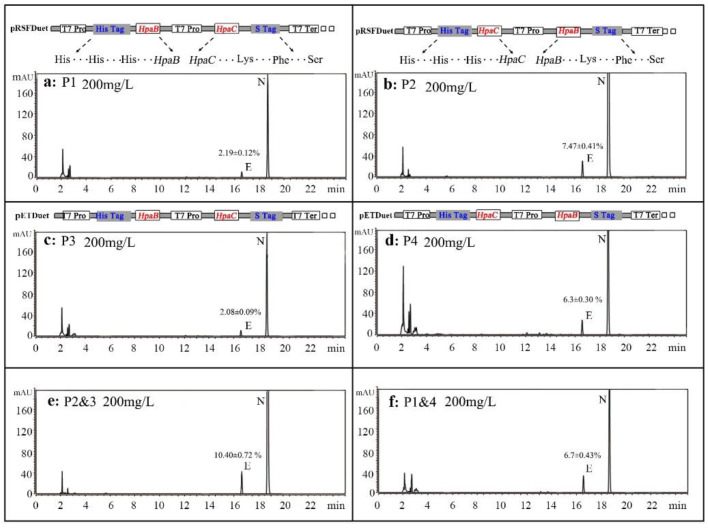
Construction strategy for all engineered Duet vectors (grey shadowed area) with HpaB and HpaC genes (colored boxes). The *ortho*-hydroxylation activities of different strains; (**a**): pRSFDuet-HpaBC (P1), (**b**): pRSFDuet-HpaCB (P2), (**c**): pETDuet-HpaBC (P3), (**d**): pETDuet-HpaCB (P4), (**e**): co-expression of P2 and P3, and (**f**): co-expression of P1 and P4. ‘His His His’ represents the three amino acid composition of His-Tag, and’ Lys Phe Ser ‘represents the label composition of S-Tag. Final substrate concentration of 200 mg·L^−1^, *n* = 3.

Several *HpaBC* expression vectors were constructed, and a two-step fermentation was performed using N as a substrate (final concentration of 200 mg·L^−1^); N and E were detected by HPLC [18]. The HPLC results showed that the strains had remarkably different *ortho*-hydroxylation activities (Figure 2). The enzyme activities of the strains carrying the P1 and P3 (containing *HpaB* in MCS-1 and *HpaC* in MCS-2) plasmids were significantly lower than those of strains carrying the P2 and P4 (containing *HpaC* in MCS-1 and *HpaB* in MCS-2) plasmids. For example, the conversion efficiency of the P2-carrying strain (7.47 ± 0.41%,15.81 ± 0.86 mg·L^−1^) was 3.81-fold higher than that of the P1-carrying strain, and that of the P4-carrying strain (6.3 ± 0.30%,13.36 ± 0.63 mg·L^−1^) was 2.86-fold higher than that of the P3-carrying strain. In addition, we also co-expressed pRSFDuet-*HpaC* (MCS-2) and pETDuet-*HpaB* (MCS-2), and the results showed very low *ortho*-hydroxylation activity (results not shown). These findings suggested that when the HpaC gene was attached to the MCS-2 locus with an S-Tag (15 amino acids) at the carboxyl-terminus, the enzyme activity could be affected.

We expressed P1&4 and P2&3 to obtain a strain with higher activity than those mentioned above. The data showed that the *ortho*-hydroxylation activity was significantly improved, and the conversion efficiency of the P2&3-carrying strain (10.40 ± 0.72%, product concentration was 22.02 ± 1.54 mg·L^−1^) was 1.39-fold higher than that of the P2-carrying strain, and that of the P1&4-carrying strain (6.7 ± 0.43%, product concentration was 14.37 ± 0.91 mg·L^−1^) was 1.07-fold higher than that of the P1-carrying strain, while the conversion efficiency of the P1&4-carrying strain was still lower than that of the P2-carrying strain. Therefore, on the basis of these observations, we have selected these two strains, including the P2&3 and P2 plasmids, and further optimized the fermentation conditions.

### 3.2. Optimization of the Induction Temperature and Substrate Delay Time

**Figure 3:**
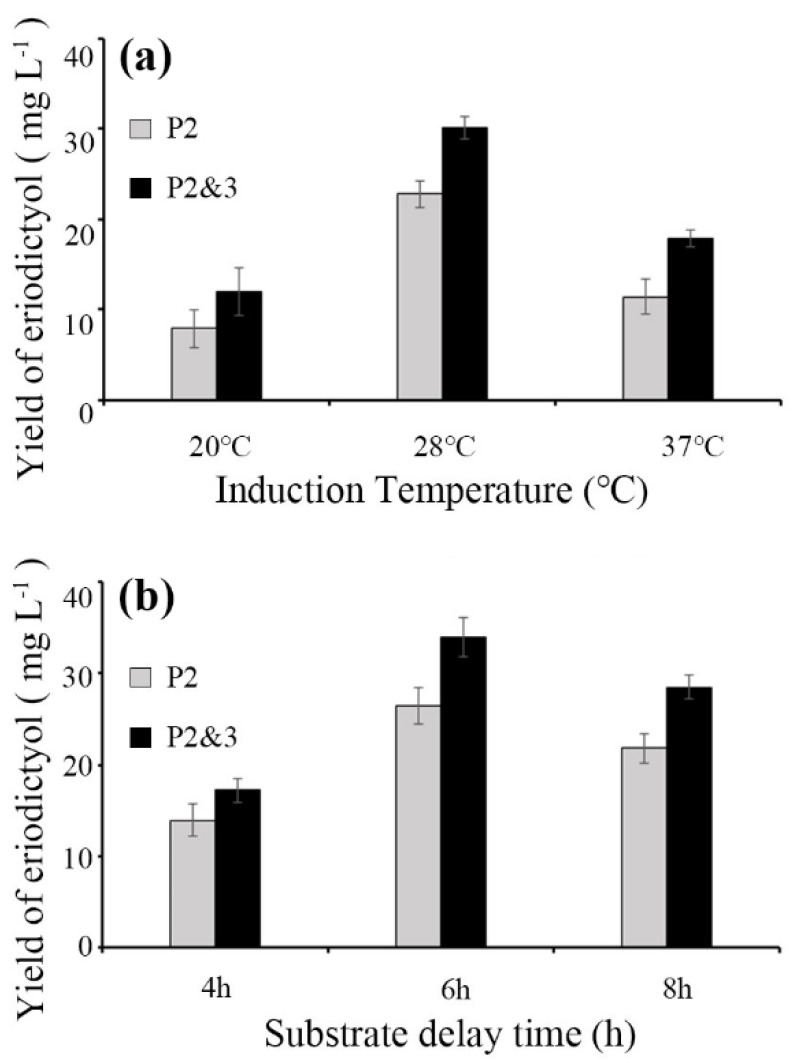
Production of E from the corresponding substrate, N. The substrate (final concentration of 200 mg·L^−1^) was added to the cell culture in LB medium. (**a**): Conversion efficiency of E at different induction temperatures. The strains were induced for 8 h at 20 °C, 28 °C or 37 °C. (**b**): Conversion efficiency of E at different substrate delay times after IPTG induction. Bacterial culture medium was induced for 4 h, 6 h or 8 h at 28 °C. Data are shown as the means ± s.d.s (*n* = 3).

To investigate the effect of culture temperature on enzyme activity, three temperatures (20 °C, 28 °C and 37 °C) were selected for fermentation (Figure 3a). The conversion efficiency of P2&3-carrying strain for E production at 37 °C was 8.46 ± 0.43% (product concentration was 17.92 ± 0.92 mg·L^−1^), which was 1.3 times higher than that produced by the P2-carrying strain at the same temperature. At the culture temperature of 28 °C, the conversion efficiency of E produced by the P2&3-carrying strain was up to 12.92 ± 0.59% (product concentration was 27.36 ± 1.26 mg·L^−1^), and the conversion efficiency of the strain carrying P2 to produce E was 10.08 ± 0.69% (product concentration was 21.35 ± 1.49 mg·L^−1^). However, the amount of product decreased, as the temperature was further reduced to 20 °C. At this temperature, the conversion efficiency of P2&3- and P2-carrying strains for E production was only 5.87 ± 1.24% and 3.45 ± 0.74% (product concentration was 12.43 ± 2.63 mg·L^−1^ and 7.31 ± 1.57 mg·L^−1^), respectively. This result indicates that in certain temperature range, the production of bioactive protein increases with the increase of temperature and reached the peak at the culture temperature of 28 °C.

To determine the optimal substrate delay time, bacterial growth medium was supplemented at 28 °C for 4 h, 6 h and 8 h after IPTG induction. The conversion efficiency of E increased gradually with increasing induction time and then reached the production peak at 6 h after IPTG induction (Figure 3b). The conversion efficiencies of the P2&3- and P2-carrying strains reached 16.47 ± 1.01% and12.50 ± 1.00% (product concentration was 33.98 ± 2.12 mg·L^−1^ and 26.48 ± 2.12 mg·L^−1^), respectively. After 8 h of induction, the conversion efficiencies of the P2&3- and P2-carrying strains decreased to 13.47 ± 00.63 and 10.29 ± 0.71% (product concentration was 28.53 ± 1.33 mg·L^−1^ and 21.81 ± 1.57 mg·L^−1^), respectively. These results show that it is possible to achieve high-density culture of recombinant bacteria and high expression of products at the optimal temperature (28 °C) and IPTG induction time (6 h). Therefore, we chose these fermentation conditions for the following study.

### 3.3. Optimization of the Substrate Concentration and Medium to Improve Catalytic Efficiency

**Figure 4:**
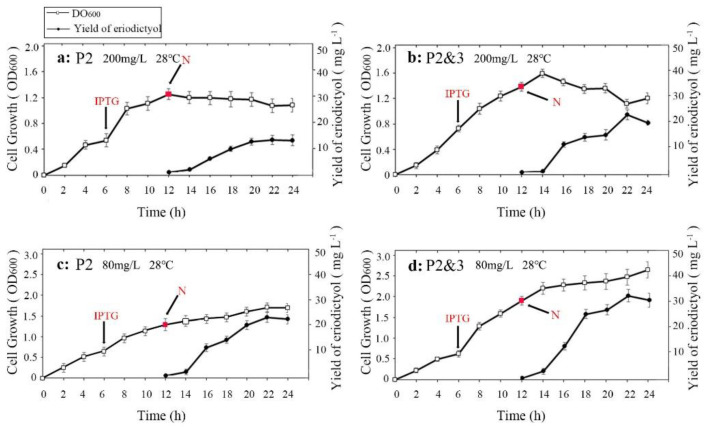
Growth curve of bacterial culture at different substrate concentrations and the conversion efficiency of E at different incubation times. The hollow boxes show the growth curve of bacterial cells, and the solid circles represent the titer of E at different incubation times. The IPTG induction time is shown by a red arrow, and the red squares indicate the substrate addition time. (**a**,**b**): Substrate (200 mg·L^−1^) in LB medium; (**c**,**d**): Substrate (80 mg·L^−1^) in LB medium. Data are shown as the means ± s.d.s (*n* = 3).

Previous studies have shown that when the medium contains high concentrations of phenylpropanoic acids or flavonoids, the growth of bacteria was significantly inhibited [20,21]. This experiment was conducted to study the effect of the initial concentration of N on the catalytic efficiency of the P2&3- and P2-carrying strains. As shown in Figure 4a,b it can be seen that at 200 mg·L^−1^ concentration of N, the cell growth rate was significantly reduced 12 h after substrate addition. Figure 4a shows that the cell concentration of the P2-carrying strain was the lowest, and the final OD_600_ (cell concentration) was only 1.201 ± 0.09, while the conversion efficiency of E was 5.81 ± 0.95% (12.32 ± 2.01 mg·L^−1^). Figure 4b also shows that the growth curve of the P2&3-carrying strain showed the most obvious downtrend, and the OD_600_ of the P2&3-carrying strain was 1.597 ± 0.06. When the initial concentration of N was 80 mg·L^−1^ (Figure 4c,d), the growth of the P2 and P2&3-carrying strains did not decrease with the addition of the substrate. The final OD_600_ of the P2-carrying strain reached 1.73 ± 0.08, and the concentration of E was 27.63 ± 2.31% (23.40 ± 1.96 mg·L^−1^). The final OD_600_ of the P2&3-carrying strain was 2.46 ± 0.18, and the highest conversion efficiency of E reached 38.49 ± 2.77% (32.60 ± 2.35 mg·L^−1^); these results indicate that a high initial concentration of N produces a certain degree of inhibition on the growth of the bacterial strains.

In addition, Figure 4 shows that as the fermentation time increased, the conversion efficiency of the product increased continuously with the cultivation time. The conversion efficiency of E could be maximized 10 h after substrate addition (this result was applicable to various concentrations of 80 mg·L^−1^ or 200 mg·L^−1^). The co-transformed strain had the highest catalytic ability, which corresponded to the results in Figure 2.

**Figure 5:**
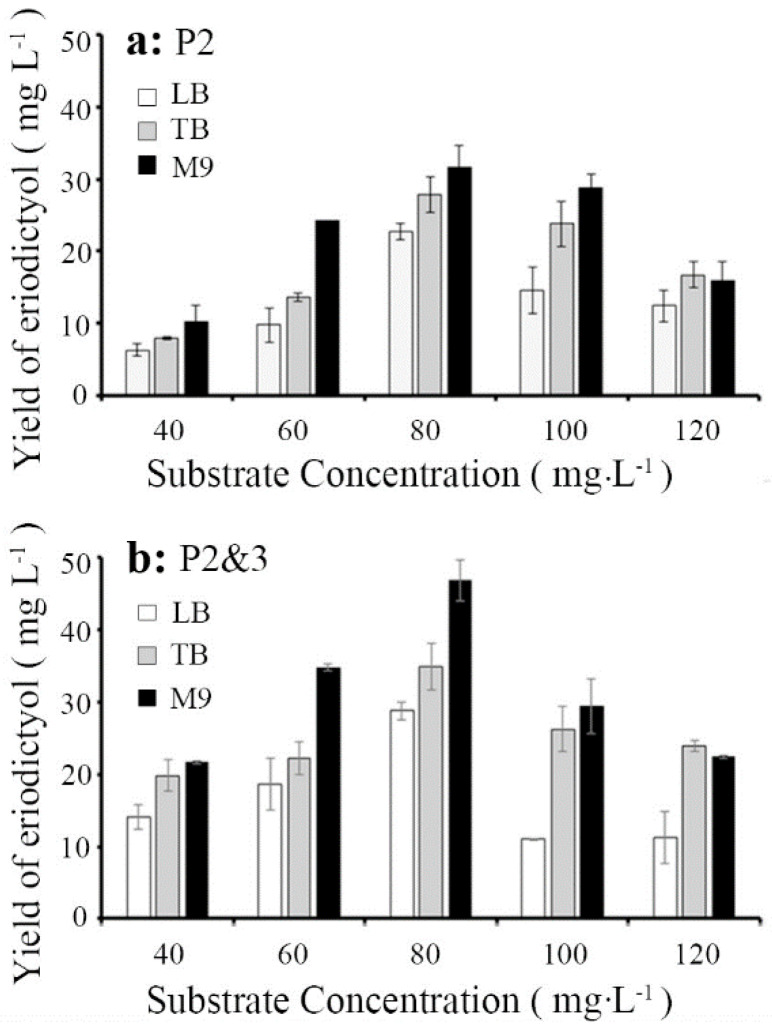
Conversion efficiency of E in different media (LB, TB and M9) and substrate concentrations (substrate concentrations from 40 mg·L^−1^ to 120 mg·L^−1^). (**a**): the conversion efficiency of E of the P2-carrying strain in LB, TB and M9 media. (**b**): the conversion efficiency of E of the P2&3-carrying strain in LB, TB and M9 media. Data are shown as the means ± s.d.s (*n* = 3).

We further studied the effects of substrate concentration and culture medium on the catalytic ability with N as a substrate based on the above results. Three types of media (including LB, TB and M9) and five substrate concentrations were selected for this study (Figure 5). The results showed that the ideal substrate concentration was 80 mg·L^−1^, and the optimal medium for E production was M9. The highest conversion efficiency of E of the P2-carrying strain was 39.58 ± 3.6% (31.67 ± 2.89 mg·L^−1^), with a final substrate concentration of 80 mg·L^−1^ in M9 medium, followed by that in TB medium (27.87 ± 2.52 mg·L^−1^), while that in LB medium was the lowest (22.72 ± 1.14 mg·L^−1^). The most exciting result was that the conversion efficiency of E produced by the P2&3-carrying strain in the conversion efficiency was up to (46.84 ± 2.85 mg·L^−1^). Hence, M9 medium and 80 mg·L^−1^ N were chosen as the optimal medium and substrate concentration, respectively, for the subsequent study.

### 3.4. Substrate Diversity Analysis of the HpaBC Complex

**Figure 6:**
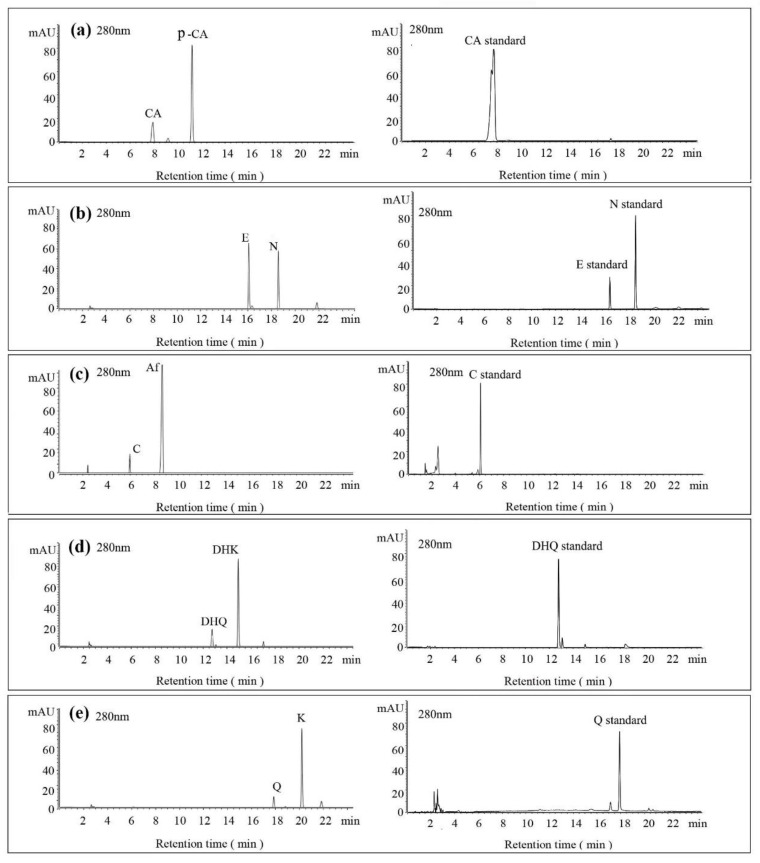
HPLC analysis of the enzymatic products of the HpaBC complex, when feeding with different substrates. HPLC chromatogram (**left**) and standard compound (**right**) analyses of the enzymatic reaction. N, E, K, Q, DHK, DHQ, C and Af were monitored at 280 nm, and *p*-CA and CA were monitored at 340 nm. The substrates and corresponding products were detected by HPLC and LC-MS. The *ortho*-hydroxylation activities of (**a**): *p*-CA to CA; (**b**): N to E; (**c**): Af to C; (**d**): K to Q; and (**e**): DHK to DHQ. Final substrate concentration of 80 mg·L^−1^, *n* = 3.

To further investigate the diversity of substrates, in addition to flavanone (N), a monohydroxylated phenolic acid (*p*-coumaric acid, *p*-CA), dihydroflavonol (DHK), flavonol (K), flavan-3-ol (afzelechin, Af) and anthocyanin (pelargonidin, PEL) were fed under the optimal conditions, and the fermentation products were detected by HPLC and LC-MS methods (Figure 6). Previous studies have suggested that the *HpaBC* complex has in vivo activity towards *p*-CA, N, and Af, with high catalytic efficiency. Our results showed that those substrates (except anthocyanin) could be converted to the corresponding *ortho*-products under the optimal conditions. Similarly, the conversion of these substrates by P2&3-carrying strains was also significantly higher than that of P2-carrying strains. The conversion rate from N to E was 58%, followed by that for Af (35.2 ± 3.14), *p*-CA (32.93 ± 2.01), DHK (23.74 ± 1.75) and K (23.84 ± 0.88), while activity on the substrate PEL was not detected (Table 2). These results provided evidence that the *HpaBC* complex had in vivo activity towards monohydroxylated flavanol and flavonol.

## 4. Discussion

Flavonoids are essential health-promoting secondary compounds produced at very low levels in plants and fungi. Metabolic engineering of flavonoids has become an extensively researched area. However, the hydroxylation on the B-ring is the bottleneck of metabolic engineering of flavonoids in *Escherichia coli* expression systems. In this paper, we used the 4-hydroxyphenylacetate 3-hydroxylase (*HpaBC*) complex to replace P450-catalyzed hydroxylation and constructed a variety of *HpaBC* expression vectors. We found that conversion activity was significantly reduced when the *HpaC* gene was attached to the MCS-2 locus with an S-Tag (15 amino acids) at the carboxyl-terminus (C-terminal). *HpaC* is a flavin reductase that catalyzes reduction of FMN to generate FMNH^−^, which is used by *HpaB* in the hydroxylation of flavonoids. Several studies have indicated that the C-terminus of *HpaC* is a self-inhibiting protein domain and that, upon binding, the effector undergoes conformational changes to allow faster flavin reduction and release [20]. We hypothesized that the C-terminus of *HpaC* connected with an expressional tag might inhibit the release of flavin, thereby inhibiting the catalytic activity of the *HpaBC* complex in vivo. In addition, by comparing the catalytic activities of different strains, we obtained two strains containing the P2 or P2&3 plasmids with increased conversion efficiency. The results showed that the co-expression of the multiple cloning site sites of *HpaB* and *HpaC* genes connected to the N-terminal of pRSFDuet vector could have a higher catalytic efficiency, but the individual plasmids will be lost in the process of culture, which inhibits the catalytic substrate process. Therefore, further studies are required to optimize the conditions for the expression of the two genes separately to improve the catalytic efficiency.

The optimum conditions, including substrate concentration, induction temperature, substrate delay time and medium, were studied in this paper using the P2- and P2&3-carrying strains. Our research found that high concentrations of the substrate (N) significantly inhibited the growth rate and conversion activity of these cells. The growth rate of the cells (the P2- and P2&3-carrying strains) was significantly reduced 12 h after adding 200 mg·L^−1^ N as the substrate. Previous reports have shown that flavonoids have significant antibacterial effects in vitro [20,21]. A low concentration of flavonoid substrates might be more conducive to an increase in the growth and activity of the recombinant strains. Through optimizing the concentration of substrate, we determined that 80 mg·L^−1^ was the optimum substrate concentration. On this basis, the conversion efficiency of the *ortho*-hydroxylated flavonoid product was the highest with 80 mg·L^−1^ N as the substrate after 6 h of induction at 28 °C in M9 medium. It is worth noting that, in the process of optimization of the optimal medium, the M9 medium was the most nutrient rich, and the catalytic efficiency of strains was the highest in M9 medium. The results of the optimal medium indicated that sufficient nutrients were needed for the culturing of the of strains, so in the industrial production process the composition of nutrients could be gradually increased to achieve the maximum efficiency. Therefore, our corresponding experiments focus on the optimization of nutrient composition in the culture medium.

**Figure 7:**
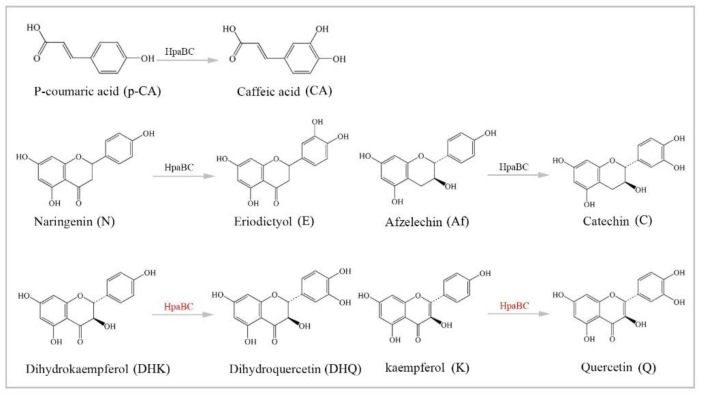
The catalytic process of HpaBC with different substrates. The red color is the first discovered catalytic activity in this study.

Substrate diversities of the *HpaBC* complex have been studied in previous studies, which have verified the catalytic activity of these complexes towards *p*-coumaric acids [21]. In addition, Jones et al. confirmed that *HpaBC* also has a catalytic effect on Af and N. In this study, we adopted optimal fermentation conditions to produce *ortho*-hydroxylated flavonoids in the *E. coli* expression system (Figure 7). Recent studies have reported that the N to E conversion rate of the recombinant HpaBC complex reached 22.12 ± 0.95% (substrate concentration was 300 mg·L^−1^, product concentration was 62.7 ± 2.7 mg·L^−1^) in vivo [18], and the maximum conversion rate was increased to 57.67 ± 3.36 (substrate concentration was 80 mg·L^−1^, product concentration was 46.84 ± 2.85 mg·L^−1^) in this study. We also obtained the corresponding products C and CA by using the corresponding substrates (Af and *p*-CA, respectively). Unfortunately, the catalytic efficiency of the recombinant HpaBC complex for *p*-CA to CA in this study has not reached 3.82 g·L^−1^ as mentioned in the literature [17], so we will further explore this catalytic condition in future studies. In addition, the activity of *HpaBC* with K and DHK as substrates was demonstrated in this paper. To the best of our knowledge, this is the first report regarding *HpaBC* production of quercetin and dihydroquercetin.

In the future, our research will focus on screening strains with tolerances to high concentrations of substrate. It is speculated that the production of *ortho*-hydroxylated flavonoids might be further increased if the optimal concentration of substrates was enhanced.

## 5. Conclusions

The hydroxylase complex, *HpaBC*, was selected to efficiently produce *ortho*-hydroxylated flavonoids in vivo. A variety of *HpaBC* expression vectors were constructed, and the corresponding products were successfully detected when feeding naringenin to the recombinant strains. The optimal culture conditions were a substrate concentration of 80 mg·L^−1^, induction temperature of 28 °C, medium of M9 medium, and substrate delay time of 6 h after IPTG induction. With the optimal conditions, the conversion efficiency of eriodictyol from P2&3-carrying strains fed naringin was up to 57.67 ± 3.36 (substrate concentration was 80 mg·L^−1^, product concentration was 46.84 ± 2.85 mg·L^−1^). The same strategy was used for catechin and caffeic acid production, and the highest conversion efficiency were 35.2 ± 3.14% and 32.93 ± 2.01%, respectively. The *HpaBC* activities towards dihydrokaempferol and kaempferol were evidenced in this paper. This study provides a feasible way to efficiently synthesize in vivo the B-ring of dihydroxy flavonoids and lays a foundation for the de novo synthesis of flavonoids by *E. coli*.

## Figures and Tables

**Table 1:** Strains and plasmids used in this study.

Strains and Plasmids	Relevant Characteristics	Source or Reference
**Plasmids**		
pRSFDuet	Double T7 promoter, ColE1 ori. Kan^R^	Novagen
pETDuet	Double T7 promoter, ColE1 ori. Amp^R^	Novagen
P1	pRSFDuet carrying (MCS-1)-HpaB and HpaC (MCS-2)	This study
P2	pRSFDuet carrying (MCS-1)-HpaC and HpaB (MCS-2)	This study
P3	pETDuet carrying (MCS-1)-HpaB and HpaC (MCS-2)	This study
P4	pETDuet carrying (MCS-1)-HpaC and HpaB (MCS-2)	This study
**Strains**		
DH5α	General cloning host	Invitrogen
BL21 * (DE3)	Host for flavonoid production and gene clones	Novagen
BL21-P1	General expression strain of pRSFDuet P1	This study
BL21-P2	General expression strain of pRSFDuet P2	This study
BL21-P3	General expression strain of pETDuet P3	This study
BL21-P4	General expression strain of pETDuet P4	This study
BL21-P2&P3	General co-expression strain of P2 and P3	This study
BL21-P1&P4	General co-expression strain of P1 and P4	This study

**Table 2:** The yield and conversion rate of *ortho*-hydroxylated flavonoids for the HpaBC complex when feeding with different substrates. The horizontal lines in the table indicate that no activities has been detected. Data are shown as the means ± s.d.s (*n* = 3).

Substrates	Products	Yield (mg·L^−1^)	Conversion Rate (%)
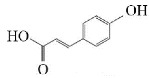 *p-*Coumaric acid	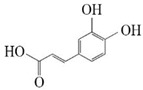 Caffeic acid	28.91 ± 1.77	32.93 ± 2.01
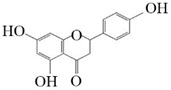 Naringenin	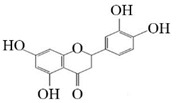 Eriodictyol	46.84 ± 2.85	57.67 ± 3.36
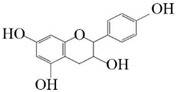 Afzelechin	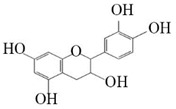 Catechin	29.81 ± 2.66	35.2 ± 3.14
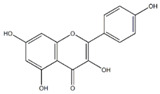 Kaempferol	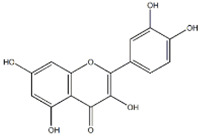 Quercetin	20.14 ± 0.75	23.84 ± 0.88
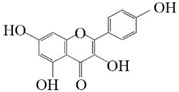 Dihydrokaempferol	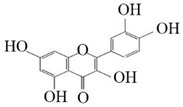 Dihydroquercetin	20.05 ± 1.48	23.74 ± 1.75
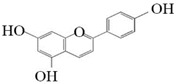 Pelargonidin	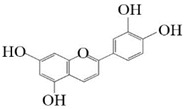 Cyanidin	_ns__	_ns__

## Data Availability

Not applicable.

## Supplementary Materials

The following are available online. Figure S1. HPLC and HPLC-MS/MS analysis of the enzymatic products of the HpaBC complex, when feeding with different substrates. Table S1. Primers of HpaB and HpaC for construction of recombinant expression plasmids.

## Sample Availability

Samples for all compounds in this article are available from the authors.
