# Supplementary material for: Optimization of the Biosynthesis of B-Ring Ortho-Hydroxy Lated Flavonoids Using the 4-Hydroxyphenylacetate 3-Hydroxylase Complex (HpaBC) of Escherichia coli
Source: Molecules. 2021 May 14;26(10):2919. doi: 10.3390/molecules26102919 (PMC8156182; doi:10.3390/molecules26102919)
Supplement: Supplementary file 1 [file molecules-26-02919-s001.zip › molecules-1181992-supplementary.pdf]

Supplement 1

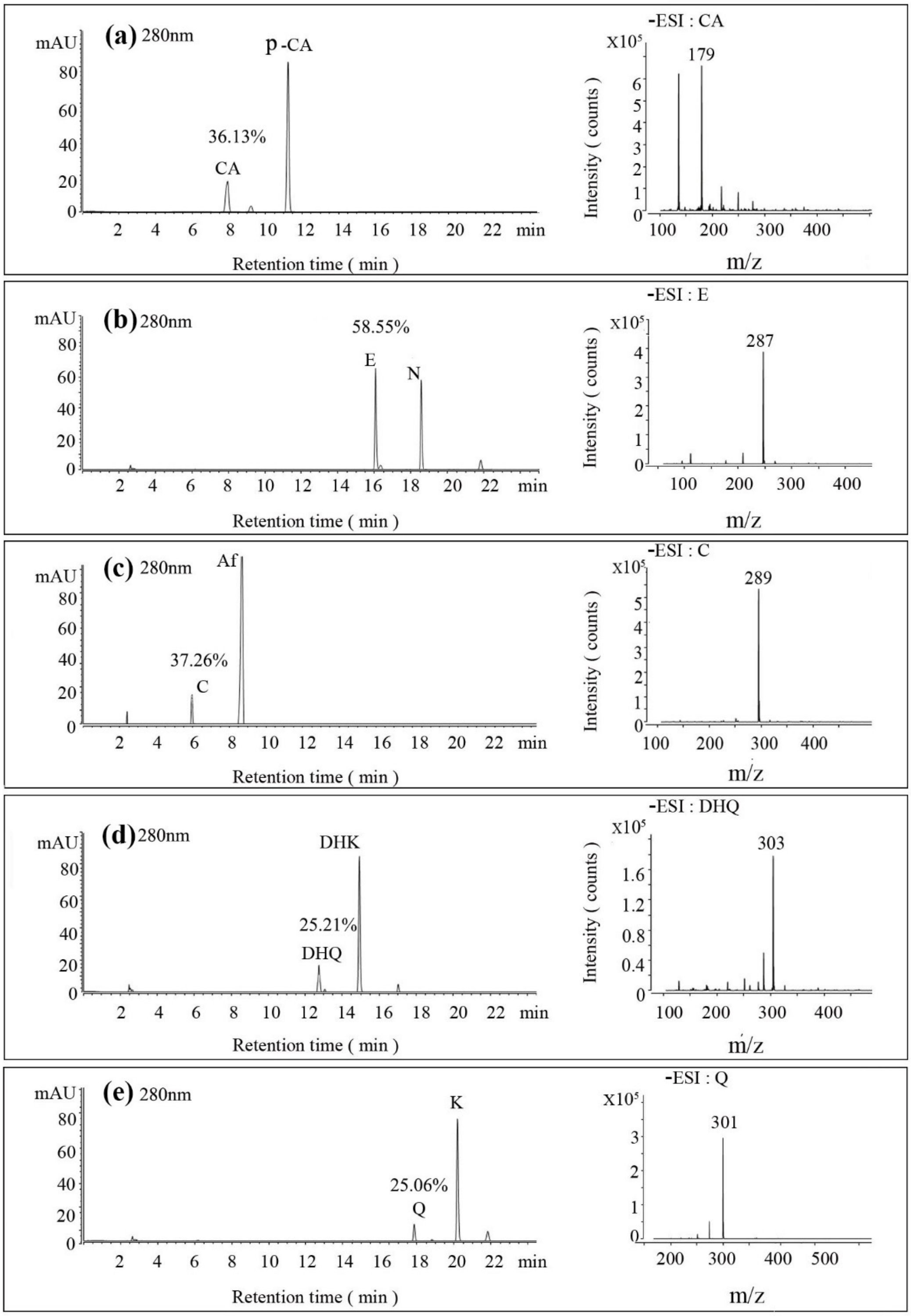

Figure S1 HPLC and HPLC-MS/MS analysis of the enzymatic products of the HpaBC complex, when feeding with different substrates. HPLC chromatogram (left) and MS/MS (right) analyses of the enzymatic reaction. N, E, K, Q, DHK, DHQ, C, Af were monitored at 280 nm, and p-CA and CA were monitored at 340 nm. The substrates and corresponding products

were detected by HPLC and LC-MS. The ortho-hydroxylation activities of (a): p-CA to CA; (b): N to E; (c): Af to C; (d): K to Q; and (e): DHK to DHQ.Final substrate concentration of 200 mg·L-1, n=3

Table S1 Primers of *HpaB* and *HpaC* for construction of recombinant expression plasmids

Primer name	Primer sequence (5'-3')	Length of PCR product (bp)
HpaB-MCS-1 (*Bam*HI)	F: ACCATCATCACCACAGCCAGGATCCGATGAAACCAGAAGATTTCCG<br>R: GGCGCGCCGAGCTCGAATTCGGATCCTTTCAGCAGCTTATCCAG	1,612
HpaC-MCS-1 (*Bam*HI)	F: ACCATCATCACCACAGCCAGGATCCGATGCAATTAGATGAACAACGC<br>R: GGCGCGCCGAGCTCGAATTCGGATCCAATCGCAGCTTCCATTTCC	562
HpaB-MCS-2 (*Nde*I, *Xho*I)	F: GGAATTCCATATGAAACCAGAAGATTTCCGCGCC<br>R: TTACCGCTCGAGTTTCAGCAGCTTATCCAGC	1,582
HpaC-MCS-2 (*Nde*I, *Xho*I)	F: GGAATTCCATATGCAATTAGATGAACAACGCCTG<br>R: TTACCGCTCGAGAATCGCAGCTTCCATTTCCAG	532
